# Role of Fucoidan on the Growth Behavior and Blood Metabolites and Toxic Effects of Atrazine in Nile Tilapia *Oreochromis niloticus* (Linnaeus, 1758)

**DOI:** 10.3390/ani11051448

**Published:** 2021-05-18

**Authors:** Abdel-Wahab A. Abdel-Warith, Elsayed M. Younis, Nasser A. Al-Asgah, Mahmoud S. Gewaily, Shaimaa M. El-Tonoby, Mahmoud A. O. Dawood

**Affiliations:** 1 Department of Zoology, College of Science, King Saud University, P.O. Box 2455, Riyadh 11451, Saudi Arabia; awarith@ksu.edu.sa (A.-W.A.A.-W.); emyounis@ksu.edu.sa (E.M.Y.); alasgah@ksu.edu.sa (N.A.A.-A.); 2 Department of Animal Production, Faculty of Agriculture, Al-Azhar University, Nasr City, Cairo 11651, Egypt; 3 Department of Anatomy and Embryology, Faculty of Veterinary Medicine, Kafrelsheikh University, Kafrelsheikh 33516, Egypt; msgewaily@vet.kfs.edu.eg; 4 Department of Animal Production, Faculty of Agriculture, Kafrelsheikh University, Kafrelsheikh 33516, Egypt; tonoby@agr.kfs.edu.eg

**Keywords:** herbicides, finfish, hepatotoxicity, renal toxicity, aquaculture, seaweeds extracts

## Abstract

**Simple Summary:**

Toxic derivatives reach the ponds and cages where fish are grown, and the continuous exposure to these contaminants proved to impair the healthy status of several finfish species. In some countries famous for cultivating rice and corn, atrazine (ATZ) is massively applied to protect plants from invaders. Many functional additives are permitted for application in the aquaculture sector as natural alternatives for chemotherapies. In this study, the toxicity impacts of ATZ and the protective role of fucoidan were investigated on the health performance of Nile tilapia. Long-term exposure to ATZ resulted in low growth rate, impaired hepato-renal function, intestinal inflammation, and oxidative stress in Nile tilapia. However, the obtained results soundly support fucoidan’s potential role to cope with the impacts of ATZ on Nile tilapia.

**Abstract:**

Waterborne herbicides are stressful agents that threaten the productivity and safety of finfish species. In this study, the toxicity impacts of atrazine (ATZ) and the protective role of fucoidan were investigated on the health performance of Nile tilapia. For 40 days, the total number of 180 Nile tilapia was assigned in four groups (triplicates, 15 fish/replicate), where the first (control) and third groups were offered the control diet, while the second and fourth groups were offered a fucoidan (FCN). Further, in the third and fourth groups, the water was mixed with atrazine (ATZ) at 1.39 mg/L daily. The growth rate, FCR, and survival rate were markedly enhanced by fucoidan but severely declined by ATZ exposure (*p* < 0.05). The morphological structure of the intestine in the control fish revealed normal structure, while fucoidan-treated groups showed eminent enhancement and branching of the intestinal villi. The intestine of ATZ-treated fish revealed deterioration and the intestinal mucosa, inflammatory cell infiltration, and separation of lining epithelium. The highest Hb, PCV, RBCs, WBCs, total protein, and albumin were observed in Nile tilapia fed fucoidan, but the worst values were seen in ATZ-intoxicated fish (*p* < 0.05). The liver-related enzymes (ALT and AST) and kidney function (urea and creatinine) showed impaired values by ATZ toxicity and were regulated by dietary fucoidan. Meanwhile, fish fed fucoidan and exposed to ATZ had lower total cholesterol and triglyceride values than fish exposed to ATZ without fucoidan feeding (*p* < 0.05). The SOD, CAT, GPx, cortisol, and glucose levels were increased in ATZ-exposed fish and reduced by fucoidan (*p* < 0.05). However, the level of malondialdehyde (MDA) was reduced in fucoidan-fed fish and increased in ATZ-exposed fish (*p* < 0.05). Altogether, dietary fucoidan is required in fish diets to alleviate the impacts of ATZ-induced toxicity.

## 1. Introduction

The aquaculture industry is still expanding to afford humanity with safe, healthy, and cheap protein sources [1]. However, the application of herbicides in the agriculture sector causes a threat to aquaculture activity. It is noteworthy that the water resources can mix with herbicides and pesticides and pollute the ecosystem in the form of waterborne toxins [2]. The toxic derivatives reach the ponds and cages where fish are grown, and the continuous exposure to these contaminants proved to impair the healthy status of several finfish species [3,4]. In some countries famous for cultivating rice and corn (e.g., Egypt), atrazine (ATZ) is massively applied to protect plants from invaders [5]. Commonly, the resulted drainage water is recycled in aquaculture ponds to culture freshwater fish species [6]. Indeed, Nile tilapia (*Oreochromis niloticus*) is the most commercial fish species consumed by many people worldwide and can grow in the agricultural leftover water [7]. The toxicity impact of herbicides, pesticides, and insecticides on the performances of aquatic animals, including Nile tilapia, is investigated in several studies [8,9]. In this regard, waterborne ATZ induces negative impacts on the performances and health status of finfish species [10]. Mainly, exposure to herbicides disrupts respiration through gills, resulting in an imbalance of metabolic functions [11]. Moreover, ATZ toxicity induces oxidative stress and the release of free radicals (ROS) involved in destroying DNA and the death of immune cells [12,13]. Therefore, genotoxicity [13], immunosuppression, oxidative stress [14], inflammation [15], hepato-renal failure [16], and low reproduction ability [17] are the significant negative impacts of ATZ toxicity in aquatic organisms. The continuous toxicity of ATZ impaired immunity and led to a high possibility of infectious attack with pathogenic bacteria. In this regard, Neamat-Allah et al. [5] stated that the continuous exposure of Nile tilapia with ATZ resulted in high susceptibility of *Aeromonas sobria* infection, resulting in a high mortality rate. 

Many functional additives are approved for application in the aquaculture sector as natural alternatives for chemotherapies [18,19]. Markedly, additives with immunostimulant properties were used in several finfish species and led to enhancements in the growth performance, health status, and welfare [20,21]. Seaweed-derived polysaccharides such as fucoidan are active substances with immunostimulant and growth-promoting activity [22,23]. In this context, dietary inclusion of fucoidan resulted in enhancing the growth performance, feed utilization, immunity, antioxidative response, and resistance against infection with bacterial and viral invaders in finfish species [24,25,26]. Fucoidan has a sufficient amount of l-fructose and sulfate ester groups known as influential immunomodulatory factors [27]. Besides, dietary fucoidan has an anti-inflammatory effect and antioxidant capacity in aquatic organisms [28]. Therefore, the inclusion of fucoidan is suggested as phytotherapy for the protection against stress induced by ATZ toxicity in finfish species.

Recently, fucoidan inclusion in the diets of African catfish (*Clarias gariepinus*) resulted in enhanced immunity and resistance against toxicity with cadmium chloride [29]. The study aimed at evaluating the protective role of fucoidan to relieve the impacts of ATZ-toxicity-induced intestinal inflammation, immunosuppression, hepato-renal injuries, and oxidative stress in Nile tilapia.

## 2. Materials and Methods

### 2.1. Fish and Feed

Two sets of experimental diets were prepared by mixing all ingredients (Supplementary File). The control diet contains the basal formulation without including fucoidan, while the second diet was prepared by mixing fucoidan (Sigma-Aldrich, St. Louis, MO, USA) at 0.8% by following Mahgoub et al. [25]. The control and fucoidan-supplemented diets were well mixed, and water and fish oil were included; then, diets were pelleted with a laboratory pelleting machine to have 2–3 mm die pellets. The pellets were kept drying in the oven at 45–50 °C, then stocked in plastic bags at 4 °C until use in the study. Nile tilapia were obtained from a local farm in the Baltim area and transported to the Fish Nutrition Laboratory, Baltim Unit, National Institute of Oceanography and Fisheries. Fish were adapted to the laboratory conditions for ten days before beginning the trial in 1000 L concrete tanks fixed with a flow-through system. During the adaptation period, fish were fed the basal diet twice daily (08:00 and 15:00). Then, fish were checked for the initial weight (16.77 ± 0.10 g), and 15 fish were stocked in 12 glass aquaria (70 L). Each group was represented with three aquaria, and each aquarium was fixed with continuous aeration while the water was replaced daily with fresh dechlorinated water.

### 2.2. Experimental Design

Four groups were assigned in this study, where each group contains three glass aquaria (15 fish/each aquarium). The first and third groups offered the control diet (control), while the second and fourth groups offered a fucoidan-supplemented diet (FCN). Half of the water used in control and FCN groups was daily exchanged with dechlorinated freshwater. In the third and fourth groups, half of the water was replaced and mixed with atrazine (ATZ) daily. The ATZ (98% purity; Sigma-Aldrich Company, St. Louis, USA) dose of toxicity was 1/5 96-h LC_50_ (1.39 mg/L) based on the findings of Neamat-Allah et al. [5]. The trial lasted for 30 days, and fish fed the respective diets twice daily (08:00 and 15:00). The water quality indices were kept at 25.21 ± 0.25 °C, 7.12 ± 0.5, 6.11 ± 0.42 mg/L, and 0.21 ± 0.01 mg/L for temperature, pH, dissolved oxygen, and total ammonia during the trial.

### 2.3. Final Sampling

All fish were starved 24 h before the final sampling. Then, fish were weighed individually (FBW, g) and counted to calculate the weight gain (WG), specific growth rate (SGR), feed conversion ratio (FCR), and survival rate.

WG = 100 × (FBW − initial weight (IBW, g))/IW (g)

SGR (percent/day) = 100 × (ln FBW (g) − ln IBW (g))/days

FCR = total dry feed intake (FI, g)/(FBW (g) − IBW (g))

Survival (%) = 100 × final fish number/initial fish number

Then fish were anesthetized with 100 mg/L tricaine methanesulfonate, and blood was collected from 3 fish/aquarium using 5 mL gauge syringes from the caudal vein. Half of the blood was kept in EDTA-heparinized tubes and immediately used for hematological analysis. The remaining blood was kept in non-heparinized tubes for serum collection. After 2 h, blood samples were centrifuged at 3000 rpm/15 min at 4 °C; then, serum was separated and kept at −20 °C for further analysis. Besides, three fish per aquarium were dissected, and their intestines were extracted to analyze the histological features.

### 2.4. Blood Analysis

White blood cell (WBC) and red blood cell (RBC) counts, as well as hemoglobin concentration (Hb), were done following standard procedure [30]. Packed cell volume (PCV) was determined by the micro hematocrit method, while the hemoglobin (Hb) concentration was determined with a spectrophotometer (Model RA 1000, Technicon Corporation, Washington, DC, USA) at 540 nm using the Blaxhall and Daisley [31] method. 

Serum total proteins and albumins were determined, according to Doumas et al. [32] and Dumas [33], while globulins content was calculated mathematically. Serum aspartate aminotransferase (AST), alanine aminotransferase (ALT), total cholesterol, creatinine, urea, uric acid, and triglycerides were detected by RA-50 chemistry analyzer (Bayer) using readymade chemicals (kits) supplied by Spinreact Co., Barcelona, Spain, following the manufacturer’s instructions. Blood glucose and cortisol levels (MG/100 mL) were measured using enzymatic PAP kits obtained from Bio-Merieux (France) [34].

Superoxide dismutase (SOD), catalase (CAT), glutathione peroxidase (GPx), and malondialdehyde (MDA) levels in serum were measured using diagnostic reagent kits following the manufacturer’s (Cusabio Biotech Co., Ltd.; Wuhan, China) instructions.

### 2.5. Histomorphology

The histological examination was adopted according to Gewaily and Abumandour [35]. The dissected intestine samples were cut into pieces of approximately 0.5 cm^3^ and fixed in neutral buffered formaldehyde 10% solution for 24 h. The samples were then dehydrated in ascending grades of alcohol, cleared with xylene, and embedded in paraffin wax. Then, 5 μm thick sections were cut using Leica rotatory microtome (RM 20352035; Leica Microsystems, Wetzlar, Germany) and stained with hematoxylin and eosin. Finally, the tissue sections were examined using a BX50/BXFLA microscope (Olympus, Tokyo, Japan).

### 2.6. Statistical Analysis

Shapiro–Wilk and Levene tests confirmed normal distribution and homogeneity of variance. The obtained data were subjected to one-way ANOVA. Differences among means were tested at *p* < 0.05 level using the Duncan test as a post-hoc test. All the statistical analyses were done via SPSS version 22 (SPSS Inc., Chicago, IL, USA).

## 3. Results

### 3.1. Growth Performance

The growth behavior (final weight (FBW), weight gain (WG), and specific growth rate (SGR)) of Nile tilapia fed dietary fucoidan and exposed to ATZ are shown in Table 1. The FBW, WG, and SGR were markedly enhanced by fucoidan but severely declined by ATZ exposure (*p* < 0.05). On the other hand, the feed conversion ratio (FCR) was meaningfully reduced by dietary fucoidan and increased by ATZ toxicity. The survival rate showed a highly significant enhancement in control and fucoidan-fed groups compared with ATZ or fucoidan/ATZ groups (*p* < 0.05). Besides, fish fed fucoidan and exposed to ATZ had a higher survival rate than fish exposed to ATZ and fed the basal diet.

### 3.2. Intestinal Health

**Figure 1:**
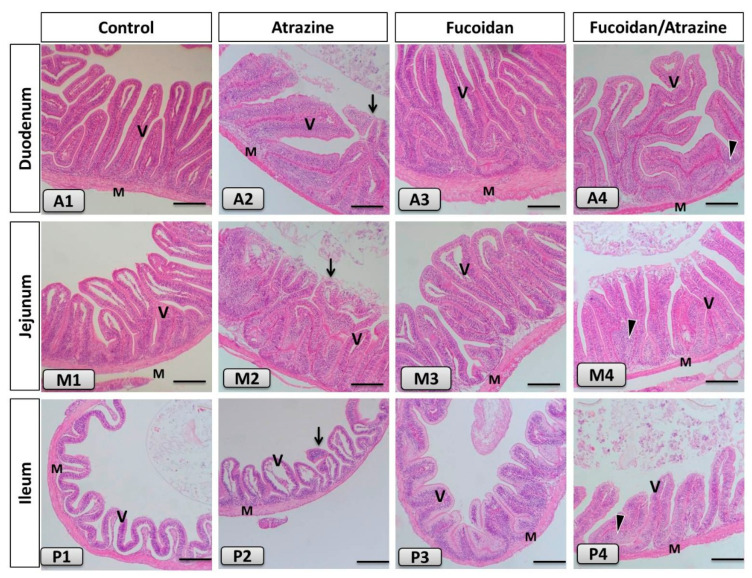
Histomicrograph of the Nile tilapia intestine of including duodenum (upper panel; **A1**–**A4**), jejunum (middle panel; **M1**–**M4**) and ileum (lower panel; **P1**–**P4**) in the control group as well as other treated (Atrazine, Fucoidan, and Fucoidan/Atrazine) groups. The histological organization in the control group as well as the Fucoidan-treated groups showed normal, intact intestinal villi (V), lamina propria sub mucosa, tunica muscularis (M) and tunica serosa with enriched growth of the intestinal villi in the Fucoidan-treated group. The intestine of atrazine-treated fish revealed destruction and inflammatory cell infiltration in the intestinal mucosa with desquamation of the lining epithelium (black arrow). The Fucoidan/Atrazine-subjected group presented apparent improvement of the intestinal villi in addition to immune cell infiltration near the base of the intestinal villi (arrowhead). Stain H&E. Bar: 100 µm.

The morphological structure of the intestine of Nile tilapia in the control fish revealed normal, intact intestinal villi, lamina propria sub mucosa, tunica muscularis and tunica serosa through the three parts: duodenum, jejunum, and ileum (Figure 1(A1,M1,P1), respectively). The histological structure in the fucoidan-treated groups (Figure 1(A3,M3,P3)) showed eminent enhancement and branching of the intestinal villi. The intestine of ATZ-treated fish revealed deterioration and the intestinal mucosa, inflammatory cell infiltration, and separation of lining epithelium (Figure 1(A2,M2,P2)). The fucoidan/ATZ-treated group exposed detectible improvement of the intestinal villi in addition to immune cell infiltration near the base of the intestinal villi (Figure 1(A4,M4,P4)).

### 3.3. Hematology

Altogether, the most significant effects of dietary fucoidan with or without ATZ toxicity were observed in hemoglobin (Hb), hematocrit (PCV), RBCs, and WBCs, while no effects (*p* ˃ 0.05) were observed in the case of RBC and WBC derivatives (Table 2). Markedly, the highest Hb, PCV, RBCs, and WBCs were observed in Nile tilapia fed fucoidan, but the worst values were seen in ATZ-intoxicated fish (*p* < 0.05). Further, fish fed fucoidan and exposed to ATZ had higher Hb, PCV, RBCs, and WBCs than fish exposed to ATZ and fed a fucoidan-free diet (*p* < 0.05).

### 3.4. Blood Biochemistry

**Figure 2:**
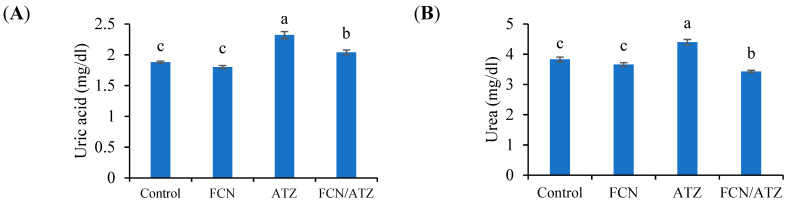

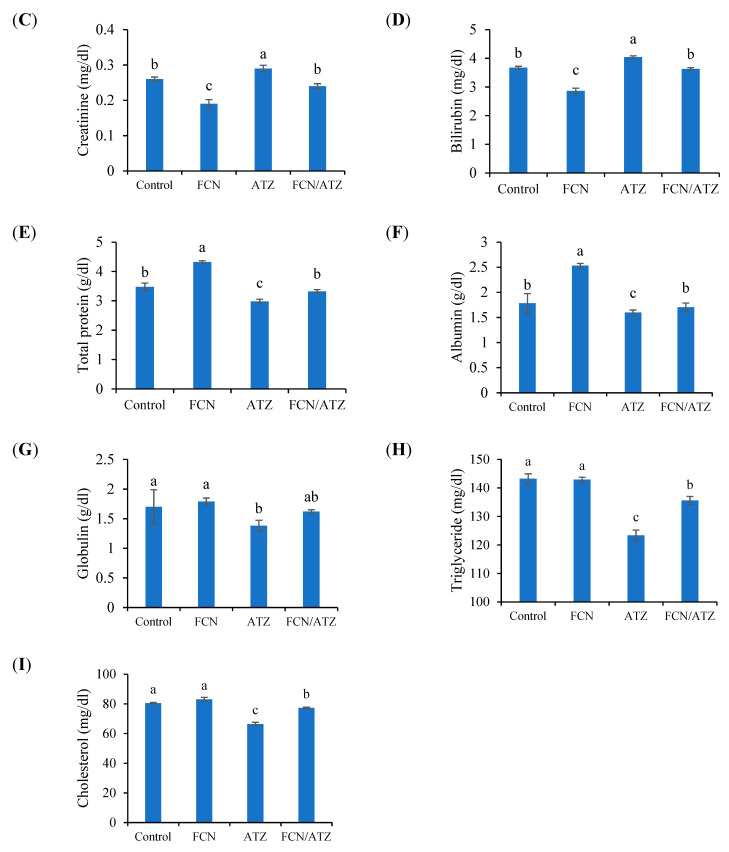
The blood biochemical traits of Nile tilapia fed fucoidan and exposed to atrazine. Bars present means ± S.E. with different letters; differ significantly (*p* < 0.05). The groups are the control, fish fed dietary fucoidan (FCN), fish fed basal diet and exposed to atrazine (ATZ), and fish fed FCN and exposed to ATZ (FCN/ATZ). Blood uric acid (**A**), urea (**B**), creatinine (**C**), bilirubin (**D**), total protein (**E**), albumin (**F**), globulin (**G**), triglycerides (**H**), and cholesterol (**I**).

The uric acid and urea levels were meaningfully higher in Nile tilapia intoxicated with ATZ with or without fucoidan than fish without ATZ toxicity (Figure 2A,B). Levels of uric acid and urea tended to decrease by fucoidan in groups of fish exposed to AZT compared with fish fed the basal diet (*p* < 0.05). The creatinine and bilirubin levels exhibited marked higher values in ATZ-exposed fish and lower values in fucoidan fed fish (*p* < 0.05), while fish exposed to ATZ and fed fucoidan had similar creatinine and bilirubin levels with regard to the control (*p* ˃ 0.05) (Figure 2C,D). The blood total protein and albumin had higher levels in fucoidan-fed fish and lower values in ATZ-exposed fish, while fish exposed to ATZ and fed fucoidan had similar total protein and albumin levels with regard to the control (*p* ˃ 0.05) (Figure 2E,F). The globulin level was obviously reduced by ATZ toxicity (*p* < 0.05) compared with fish fed the basal and fucoidan diets without ATZ toxicity (Figure 2G). The levels of triglycerides and total cholesterol were significantly reduced by ATZ toxicity (*p* < 0.05) compared with fish fed the basal and fucoidan diets without ATZ toxicity (Figure 2H,I). Meanwhile, fish fed fucoidan and exposed to ATZ had lower values than fish exposed to ATZ without fucoidan feeding (*p* < 0.05).

**Figure 3:**
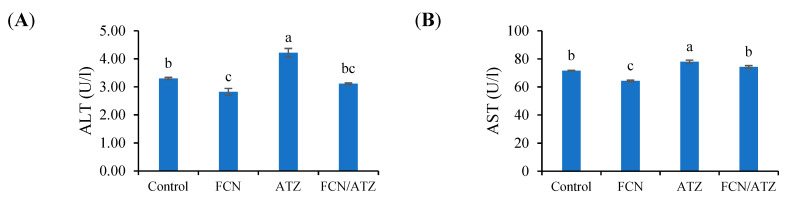
The liver-related enzymes of Nile tilapia fed fucoidan and exposed to atrazine. Bars present means ± S.E. with different letters; differ significantly (*p* < 0.05). The groups are the control, fish fed dietary fucoidan (FCN), fish fed basal diet and exposed to atrazine (ATZ), and fish fed FCN and exposed to ATZ (FCN/ATZ). Serum alanine aminotransferase (ALT) (**A**) and aspartate aminotransferase (AST) (**B**).

The activities of liver enzymes (ALT and AST) were higher in ATZ-exposed fish and lower values in fucoidan fed fish (*p* < 0.05), while fish exposed to ATZ and fed fucoidan had similar values of ALT and AST with regard to the control (*p* ˃ 0.05) (Figure 3A,B).

**Figure 4:**
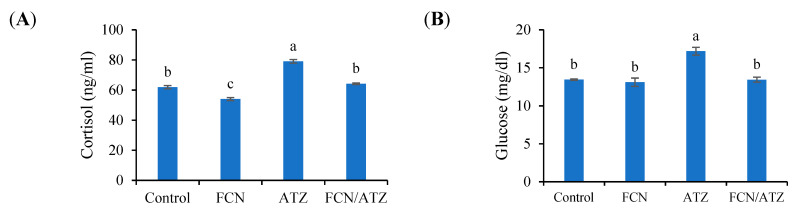
The blood stress factors of Nile tilapia fed fucoidan and exposed to atrazine. Bars present means ± S.E. with different letters; differ significantly (*p* < 0.05). The groups are the control, fish fed dietary fucoidan (FCN), fish fed basal diet and exposed to atrazine (ATZ), and fish fed FCN and exposed to ATZ (FCN/ATZ). Blood cortisol (**A**) and glucose (**B**).

The cortisol level manifested higher values in ATZ-exposed fish and lower values in fucoidan fed fish (*p* < 0.05), while fish exposed to ATZ and fed fucoidan had similar cortisol levels with regard to the control (*p* ˃ 0.05) (Figure 4A). Besides, the glucose level showed a higher value in the ATZ-exposed group than the other groups (*p* < 0.05) (Figure 4B).

### 3.5. Antioxidative Status

**Figure 5:**
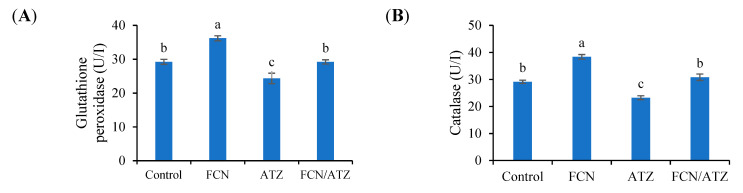

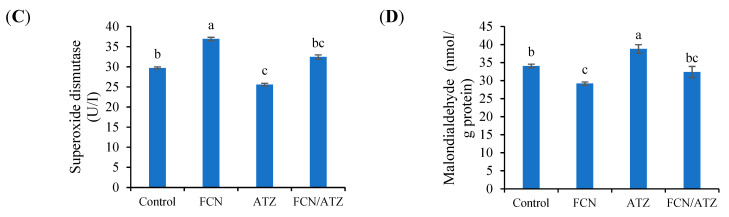
The blood antioxidative traits of Nile tilapia fed fucoidan and exposed to atrazine. Bars present means ± S.E. with different letters; differ significantly (*p* < 0.05). The groups are the control, fish fed dietary fucoidan (FCN), fish fed basal diet and exposed to atrazine (ATZ), and fish fed FCN and exposed to ATZ (FCN/ATZ). Glutathione peroxidase (**A**), catalase (**B**), superoxide dismutase (**C**), and malondialdehyde (**D**).

The activities of SOD, CAT, and GPx were increased in fucoidan-fed fish and decreased in ATZ-exposed fish (*p* < 0.05), while fish exposed to ATZ and fed fucoidan had similar SOD, CAT, and GPx activities with regard to the control (*p* ˃ 0.05) (Figure 5A–C). However, the level of malondialdehyde (MDA) was reduced in fucoidan-fed fish and increased in ATZ-exposed fish (*p* < 0.05), while fish exposed to ATZ and fed fucoidan had similar MDA level with regard to the control (*p* ˃ 0.05) (Figure 5D).

## 4. Discussion

Aquatic organisms require optimum conditions for regular growth and wellbeing [36]. However, unstable rearing conditions, especially low water quality, could affect fish’s health condition and thereby the growth performance and productivity [37]. Rearing water can be polluted by waterborne herbicides resulting from the agriculture sector and directly impair fish’s health condition [15]. According to the literature, atrazine’s pollution resulted in hazardous effects on fish such as immunosuppression, oxidative stress, inflammation, and high acceptability to infection [13,14,16]. For a long time, antibiotics were applied to enhance aquatic animals’ immunity [38], but it is no longer allowed in many countries, and other natural immunostimulants are suggested [39]. Seaweed extracts are practically applied in aquaculture and are evidenced as natural growth promotors and immunostimulants [22,23]. 

The results show the enhanced growth rate (final weight, weight gain, and specific growth rate) and feed conversion ratio of Nile tilapia delivered fucoidan compared with other groups. Further, fish fed fucoidan and exposed to ATZ had higher growth rates than fish exposed to ATZ without fucoidan feeding. The results are comparable with those of Mahgoub et al. [25], who illustrated that Nile tilapia fed fucoidan had enhanced growth performance and feed utilization. Additionally, the inclusion of fucoidan in the diets of red sea bream (*Pagrus major*) [40], gibel carp (*Carassius auratus*) [41], and *Labeo rohita* [26] enhanced the growth performance. The enhanced growth performance is possibly attributed to the high feed utilization in Nile tilapia delivered fucoidan for 30 days. The enrichment of fucoidan in tilapia diets probably improved the intestinal health and digestion capacity through intestinal villi [41]. Dietary fucoidan may result in increased villi height and length and thereby area of nutrient digestion and absorption [25]. Besides, fucoidan supplementation could enhance the diversity of intestinal microbiota leading to a high abundance of beneficial microorganisms involved in the secretion of digestive enzymes [22,23]. Consequently, feeds can be digested efficiently in fish intestines and enhance the delivery of nutrients to the bloodstream and the entire body [42]. The results also showed enhanced intestinal features (height, length, and internal distance) in Nile tilapia fortified with fucoidan, but the morphometrical indices were depressed by ATZ toxicity. These results explain that fucoidan is involved in enhancing intestinal health and absorption capacity. In a similar sense, the inclusion of fucoidan in Nile tilapia [25] and gibel carp [43] diets resulted in enhanced intestinal morphometrical indices. Nevertheless, ATZ toxicity induced abnormal and inflammatory features in the intestine of Nile tilapia, explaining the lowered feed efficiency and growth performance. In the same line, exposure to pesticides resulted in impaired intestinal health in Nile tilapia [44]. The damaged intestinal features and inflammation are probably attributed to the oxidative stress induced by ATZ exposure [5,12]. The survival rate is also lowered by ATZ toxicity and showed the best value in the group delivered fucoidan. The enhanced survival rate is correlated with the improved health condition of Nile tilapia fed fucoidan. On the other hand, the lowered survival rate in fish exposed to ATZ for 30 days is associated with inflammation, oxidative stress, and immunosuppression.

The hematological indices are recommended for evaluating the metabolic and immunological status of fish suffering from toxic and pathogenic stressors [45,46]. The levels of hemoglobin (Hb) and red blood cells (RBCs) indicate the anemic and respiration capacity of blood cells, whereas hematocrit (PCV) and white blood cell (WBCs) levels show the immunological status of fish [47]. The present study shows that Nile tilapia fed fucoidan had the best health condition, while fish exposed to ATZ had the worst health status, as indicated by the levels of Hb, PCV, RBCs, and WBCs. Interestingly, the control fish and fish fed fucoidan and exposed to ATZ had similar Hb, PCV, WBC, and RBC levels. The results illustrate that fucoidan raised the immunological status and metabolic function in Nile tilapia to cope with the adverse impacts of ATZ. However, exposure to ATZ result in impaired Hb, PCV, RBCs, and WBCs, probably due to oxidative stress and low metabolic function [15]. Similarly, chirruh snowtrout (*Schizothorax esocinus*) [45,48] exposed to ATZ had deteriorated hematological traits while fish fed fucoidan had enhanced hematological indices.

Biochemical blood factors are diagnostic tools for evaluating the metabolic rate (blood protein and globulin), kidney function (creatinine, bilirubin, and uric acid), liver function (albumin, ALT, AST, and ALP), and stress indices (cortisol and glucose) of the organism [49,50]. Under toxicity conditions, the levels of these indices may deteriorate, referring to dysfunction of vital organs involved in renal, metabolic, antioxidative, and immunity roles in the fish body [6]. The results show high levels of urea, creatinine, ALT, AST, triglyceride, and total cholesterol in fish exposed to ATZ. At the same time, dietary fucoidan mitigated the impacts of ATZ on the mentioned blood biochemical indices. The toxicity with ATZ showed disruption in liver and kidney function in Nile tilapia devoted to the adverse impact of ATZ-induced inflammation on the structure and function of tissue cells leading to irregular secretion of enzymes and lipid droplets [12,51,52]. Besides, the levels of total protein, albumin, and globulin were lowered by ATZ toxicity, but dietary fucoidan regulated the levels of blood proteins. Fucoidan supplements are known for their functionality as metabolic and immunological mediators involved in enhancing the health status and wellbeing of aquatic organisms [24]. Further, fucoidan regulates the metabolic function and level of proteins and nutrients in the blood, leading to high proteins and immune-related factors [53]. Concurrent with the present study, dietary fucoidan regulated the metabolites, liver enzymes, and kidney-related factors in Nile tilapia [54].

Waterborne toxins are stressful agents involved in raising the stress-related factors (e.g., cortisol and glucose) [55]. Fish suffering from stress produce high cortisol levels, which led to glucose secretion from the liver via the gluconeogenesis process to afford the fish body with sufficient energy required to counteract stressful impacts [56]. Concerning the present study, Nile tilapia exposed to ATZ had higher cortisol and glucose levels than those fed fucoidan, indicating the anti-stressful influence of fucoidan. In agreement, Nascimento et al. [57] stated increased glucose and cortisol levels in the blood of *Prochilodus lineatus* exposed to ATZ. In another study, Yang et al. [58] reported that yellow catfish fed dietary fucoidan had lowered glucose and cortisol levels.

Toxicity with herbicides and pesticides is one of the main reasons for oxidative stress in fish [59]. Short- and long-term exposure to waterborne toxins causes degeneration of free radicals (ROS), leading to lipid peroxidation and cell damage in the fish body [12]. Consequently, fish secrete enzymatic and non-enzymatic factors to deactivate the oxidation and protect cells from ROS attack [10]. This study shows activated SOD, CAT, and GPx levels and reduced MDA levels in fish fed fucoidan. However, ATZ toxicity induced lowered SOD, CAT, and GPx and high MDA levels, referring to oxidative stress. Overoxidation is the main reason for inflammation and immunosuppression in the entire body, tissues and organs [15]. Besides, oxidative stress is involved in the dysfunction of fish’s vital functions, leading to low metabolic and anti-stress ability [60]. Therefore, the reduction of growth rate, feed utilization, impaired renal and liver function in this study can be attributed to the adverse impact of ATZ toxicity. In the same line, Toughan et al. [15], Paulino et al. [12], and Neamat-Allah et al. [5] reported reduced antioxidative capacity in common carp (*Cyprinus carpio* L.), *P. lineatus*, and Nile tilapia exposed to ATZ. Nonetheless, dietary fucoidan resulted in enhanced antioxidative capacity in Nile tilapia [25], yellow catfish (*Pelteobagrus fulvidraco*) [58], and red sea bream (*Pagrus major*) [40]. The antioxidative capacity of fucoidan is related to the inhibition of ROS by removing hydroxyl and superoxide radicals and reducing lipid peroxidation [61]. Furthermore, dietary fucoidan is proved as an anti-inflammatory agent devoted to its role in inhibiting the ROS in vivo [28].

## 5. Conclusions

It can be assumed that dietary fucoidan is required for protecting Nile tilapia from atrazine waterborne toxicity. Long-term exposure to atrazine resulted in low growth rate, impaired hepato-renal function, intestinal inflammation, and oxidative stress in Nile tilapia. However, the obtained results soundly support fucoidan’s potential role to cope with the impacts of atrazine on Nile tilapia. Further future studies are required to clarify the functionality of fucoidan by detecting the proteomic and microbiome effects.

## Figures and Tables

**Table 1:** Growth performance of Nile tilapia fed fucoidan and exposed to atrazine.

	Control	FCN	ATZ	FCN/ATZ
IBW (g)	16.38 ± 0.24	16.77 ± 0.10	16.33 ± 0.09	16.31 ± 0.08
FBW (g)	33.90 ± 0.29 b	41.90 ± 1.44 a	27.90 ± 1.47 c	32.49 ± 0.67 b
WG (%)	106.96 ± 1.97 b	149.95 ± 8.84 a	70.74 ± 8.15 c	99.23 ± 5.05 b
SGR (%/day)	2.42 ± 0.03 b	3.05 ± 0.12 a	1.78 ± 0.16 c	2.30 ± 0.09 b
FI (g/fish)	27.78 ± 0.37	30.49 ± 1.22	30.11 ± 2.89	27.76 ± 1.02
FCR	1.59 ± 0.03 b	1.22 ± 0.02 c	2.62 ± 0.08 a	1.72 ± 0.07 b
Survival (%)	97.78 ± 2.22 a	100.00 ± 0.00 a	80.00 ± 3.85 c	86.67 ± 3.85 b

Means ± S.E. in the same row with different letters; differ significantly (*p* < 0.05). The groups are the control, fish fed dietary fucoidan (FCN), fish fed basal diet and exposed to atrazine (ATZ), and fish fed FCN and exposed to ATZ (FCN/ATZ).

**Table 2:** Hematological profile of Nile tilapia fed fucoidan and exposed to atrazine.

Item	Control	FCN	ATZ	FCN/ATZ
Hb (g/100 mL)	11.90 ± 0.15 b	12.49 ± 0.19 a	10.01 ± 0.18 d	11.05 ± 0.11 c
RBCs (×10^6^/mm^3^)	3.72 ± 0.11 b	4.17 ± 0.04 a	3.14 ± 0.06 c	3.64 ± 0.04 b
pcv (%)	36.33 ± 0.33 b	39.00 ± 0.58 a	32.67 ± 0.88 c	36.33 ± 0.88 b
MCV (µm^3^/cell)	97.71 ± 2.28	93.49 ± 2.28	103.95 ± 2.56	99.96 ± 3.10
MCH (pg/cell)	32.00 ± 0.86	29.93 ± 0.60	31.87 ± 0.87	30.39 ± 0.03
MCHC (%)	32.74 ± 0.18	32.02 ± 0.39	30.66 ± 0.45	30.46 ± 0.90
WBCs (×10^3^/mm^3^)	39.19 ± 0.50 b	41.39 ± 0.52 a	35.67 ± 0.57 c	38.48 ± 0.69 b
Heterophil (%)	7.00 ± 0.00	7.67 ± 0.33	6.33 ± 0.33	6.67 ± 0.33
Lymphocyte (%)	82.67 ± 0.67	85.00 ± 0.58	80.33 ± 0.33	82.67 ± 0.33
Monocyte (%)	6.00 ± 0.00	3.67 ± 0.33	7.33 ± 0.33	6.00 ± 0.00
Eosinophil (%)	1.67 ± 0.33	1.67 ± 0.33	3.00 ± 0.00	2.33 ± 0.33
Basophil (%)	2.67 ± 0.33	2.00 ± 0.00	3.00 ± 0.00	2.33 ± 0.33

Means ± S.E. in the same row with different letters; differ significantly (*p* < 0.05). The groups are the control, fish fed dietary fucoidan (FCN), fish fed basal diet and exposed to atrazine (ATZ), and fish fed FCN and exposed to ATZ (FCN/ATZ).

## Supplementary Materials

The following are available online at https://www.mdpi.com/article/10.3390/ani11051448/s1.
