# Supplementary material for: Role of Fucoidan on the Growth Behavior and Blood Metabolites and Toxic Effects of Atrazine in Nile Tilapia Oreochromis niloticus (Linnaeus, 1758)
Source: Animals (Basel). 2021 May 18;11(5):1448. doi: 10.3390/ani11051448 (PMC8157872; doi:10.3390/ani11051448)
Supplement: Supplementary file 1 [file animals-11-01448-s001.zip › animals-1182886-supp.pdf]

Table S1. Basal diet and proximate chemical composition (on dry matter basis)

Ingredient	(%)	Chemical composition	(%)
Fish meal (65%)	10	Crude protein	30.14
Soybean meal (44%)	36	Crude lipids	6.98
Gluten	5	Ash	7.57
Wheat bran	12	Crude fibers	5.45
Rice bran	10	Gross energy (MJ/kg)[2]	18.44
Yellow corn	12		
Wheat flour	8.92		
Fish oil	3		
Vitamin and mineral mix[1]	2		
Dicalcium phosphate	1		
Vitamin C	0.08		

[1]Vitamin and mineral mixture (per kg premix): vitamin A (3300 IU), vitamin D_3 (410 IU), vitamin B_1 (133 mg), vitamin B_2 (580 mg), vitamin B_6 (410 mg), vitamin B_{12} (50 mg), biotin (9330 mg), colin chloride (4000 mg), vitamin C (2660 mg), inositol (330 mg), para-amino benzoic acid (9330 mg), niacin (26.60 mg), pantothenic acid (2000 mg), manganese (325 mg), iron (200 mg), copper (25 mg), iodine, cobalt (5 mg).

[2]Gross energy was calculated based on the values of values for protein, lipid, and carbohydrate as 23.6, 39.5 and 17.2 KJ/g, respectively.
